# Novel Natural Inhibitors of CYP1A2 Identified by *in Silico* and *in Vitro* Screening

**DOI:** 10.3390/ijms12053250

**Published:** 2011-05-18

**Authors:** Ruixin Zhu, Liwei Hu, Haiyun Li, Juan Su, Zhiwei Cao, Weidong Zhang

**Affiliations:** 1 Department of Natural Medicinal Chemistry, School of Pharmacy, Second Military Medical University, Shanghai 200433, China; E-Mails: rxzhu@tongji.edu.cn (R.Z.); liwei8192@yahoo.com.cn (L.H.); kotorinun@126.com (H.L.); juansu_2008@126.com (J.S.); 2 School of Life Sciences and Technology, Tongji University, 1239 Siping Road, Shanghai 200092, China; 3 Shanghai Center for Bioinformation Technology, 100 Qinzhou Road, Shanghai 200235, China; 4 School of Pharmacy, Shanghai Jiao Tong University, Shanghai 200030, China

**Keywords:** CYP1A2, pharmacophore, docking, herb–drug interaction

## Abstract

Inhibition of cytochrome P450 (CYP) is a major cause of herb–drug interactions. The CYP1A2 enzyme plays a major role in the metabolism of drugs in humans. Its broad substrate specificity, as well as its inhibition by a vast array of structurally diverse herbal active ingredients, has indicated the possibility of metabolic herb–drug interactions. Therefore nowadays searching inhibitors for CYP1A2 from herbal medicines are drawing much more attention by biological, chemical and pharmological scientists. In our work, a pharmacophore model as well as the docking technology is proposed to screen inhibitors from herbal ingredients data. Firstly different pharmaphore models were constructed and then validated and modified by 202 herbal ingredients. Secondly the best pharmaphore model was chosen to virtually screen the herbal data (a curated database of 989 herbal compounds). Then the hits (147 herbal compounds) were continued to be filtered by a docking process, and were tested *in vitro* successively. Finally, five of eighteen candidate compounds (272, 284, 300, 616 and 817) were found to have inhibition of CYP1A2 activity. The model developed in our study is efficient for *in silico* screening of large herbal databases in the identification of CYP1A2 inhibitors. It will play an important role to prevent the risk of herb–drug interactions at an early stage of the drug development process.

## 1. Introduction

During the last decade, North America and Europe have witnessed a drastic growth in the use of herbal medicine. In a comprehensive review on the usage of complementary and alternative medicine, 16% of prescription drug users are reported to consume herbal supplements [1], two-thirds of women used herbs for perimenopausal symptoms, 45% of parents gave their children herbal treatments and 45% of pregnant women tried herbal remedy [2]. Meanwhile, some reports have indicated that more than 60% of patients use their herbal medicine with conventional medicines as usual [3]. Because some of constituents in herbal products may be substrates, inhibitors and/or inducers of the cytochrome P450 enzymes (CYPs) [4,5], thus could have an impact on the pharmacokinetics of a co-administered drug metabolized by the CYP enzymes, which will probably lead to the herb–drug interaction. The most well-known example of an herb–drug interaction is St John’s wort, which could cause the clearance of CYP1A2 substrates such as theophylline [6]. Clinically significant interactions were also reported from the use of grapefruit juices in calcium antagonists, antihistamine and benzodiazepines treatment [7–9]. Nowadays, the interaction of drugs with herbal medicines has become a significant safety concern, especially for drugs with narrow therapeutic indices.

Among all of these cytochrome P450 enzymes, CYP1A2 should attract more attention since it is one of the key enzymes with the important role in the metabolic clearance of 5% of currently marketed drugs [10]. Also it is involved in a number of clinical herb–drug interactions [11–13]. When herbal medicines are used with western medicines metabolized by CYP1A2 at the same time, it is necessary to know whether this herb medicine will inhibit CYP1A2 or not. This means that the prediction of herb–drug interactions *in vitro* is important and thus many herbal medicines were tested *in vitro* by scientists [14–16]. However, the number of herbal medicines is large. Traditional screening technologies such as testing each herbal medicine to enzyme *in vitro* or *in vivo* would not only be costly, but also inefficient. Recently, several attempts in the application of computational models for CYP1A2 ligand binding have been reported, reflecting the desire of early identification of CYP1A2 inhibitors [17–22]. Taesung Moon *et al*. [23] pioneered investigations on the quantitative structure-activity relationship (QSAR) of flavonoids and their derivatives. However, their samples were only flavonoids, which were not representative of all herb ingredients and fail to accurately predict the inhibitory potencies of structurally unrelated herb ingredients.

In order to better predict herb–drug interaction as well as select rapid inhibitors from herbal ingredients, a combinational pharmacophore model were presented to virtually screen the herb data before those candidate compounds were tested *in vitro* to determine their inhibitory effect on CYP1A2. The model developed here is efficient for virtual screening of large herbal databases for identification of CYP1A2 inhibitors, and it will play an important role to prevent the risk of herb–drug interactions at an early stage of the drug development process.

## 2. Results and Discussion

### 2.1. Pharmacophore Models

**Figure 1.**
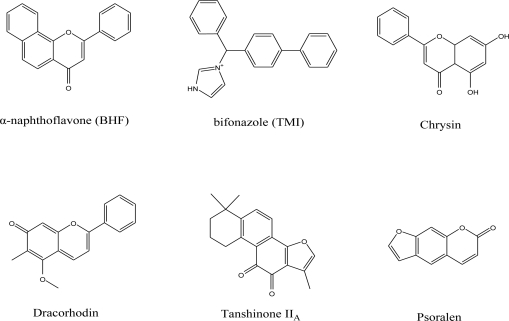
Molecular structure of the template molecules used in this work.

**Figure 2.**
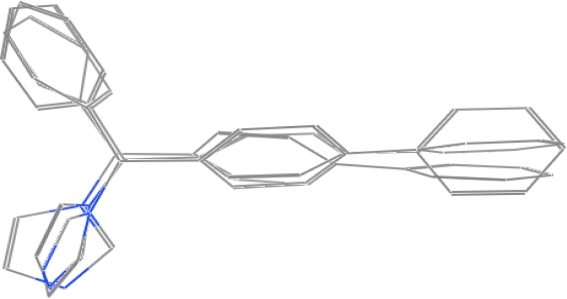
The molecular structure of selected template by superposing three bifonazole in three different conformations.

**Figure 3.**
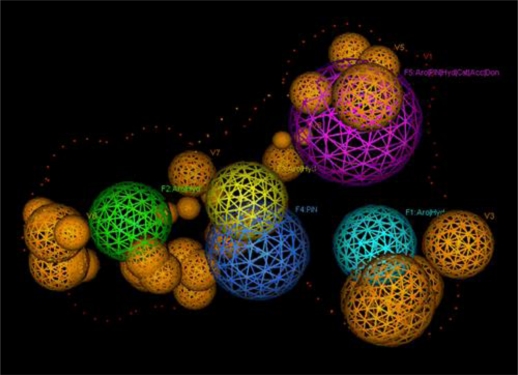
The final pharmacophore of CYP1A2. F1–F3: Aro|Hyd; F4: PiN; F5: Aro|PiN|Hyd|Cat|Acc|Don; V1: Exterior Volume; V2–V8: Excluded Volume.

For the pharmacophore screening, the key step was to choose a good template molecule. In this study, several template molecules (Figure 1) could be obtained to generate the pharmacophore: (1) the substrates extracted from complex structures of CYP1A2 and its homologous enzymes; and (2) inhibitors reported in the literature [24]. Different template molecules based on individual or integrated information above were used to generate the pharmacophores. Then up to 202 different herb integrants tested *in vitro* by our group were used as the test dataset (supplement Table 2). The molecular structure of selected template was shown in Figure 2. Finally, the pharmacophore model was obtained (Figure 3). The true positive rate and true negative rate of the best pharmacophore model were 84.6% (11/13) and 86.8% (164/189), respectively. Other results of different pharmacophore models are also shown in Table 1 as a comparison.

There were still disputes on how to superpose these template molecules used to build the pharmacophore [25]. It is known that there are two opinions on the active conformation [26–28]; some people prefer to optimize the energy of these template molecules and obtain the conformation with minimum energy. While another group of people believe that the docking conformation is the active conformation and these template molecules extracted from the crystal structure should be superposed directly. Our results have shown that the latter opinion is more reasonable compared with the former one [29].

By observing the complex crystal structure of CYP1A2 and its homologous enzymes, we found that not only different substrates could exist in the same acceptor, but also different conformations of one substrate could exist within one complex crystal structure. It is obvious that superposing the different conformations of one substrate appearing in one complex crystal structure will be the best option, since these conformations were not only the true active conformation, but also reduces errors commonly caused by trying different crystal structures, measurements and analysis conditions. As a validation, the results shown in Table 1 were proved by Zhao *et al.* recently [24].

In addition, our work also indicated that it was important to collect some negative data in the building of pharmacophore, since excluded volume of the pharmacophore was built on the negative data. Also the building of excluded volume is the key to increase the true negative rate. However, this step was often ignored by former research groups.

Finally, 147 hits were filtered out by the selected pharmacophore model from 989 compounds, which were separated from various herbs collected in our group. Formerly, compounds in Chinese Nature Products Database (CNPD v.2004.1) [30] were also screened by using this pharmacophore model. Unfortunately, this research had to be abandoned because hits in CNPD were unavailable.

### 2.2. Docking Results

Admittedly, two challenges in the field of molecular docking still exist: (1) ligand placement in active site, and (2) scoring of docked poses [31,32]. However, compared with the semi-quantitative method of the pharmacophore model, molecular docking, as one of the quantitative methods, is better for prioritizing the hits with the help of deriving stable docking parameters and combing. Recently, the work of Yu Chen and Brian K. Shoichet [33] reinforced more confidence to docking results.

The purpose of the dock application is to search for favorable binding configurations between small to medium-sized ligands and a not too flexible macromolecular target, which is usually a protein. For each ligand, a number of configurations called poses are generated and scored in an effort to determine favorable binding modes. Optionally, poses can be constrained to fit a pharmacophore query. The top scoring poses are written to a database for further analysis.

In addition, 147 compounds formerly filtered from the pharmacophore model were docked into the active site of CYP1A2. For comparison, docking was run twice and results are presented in supplement Table 1. It can be seen that this set of docking parameters almost produced stable docking results.

**Figure 4.**
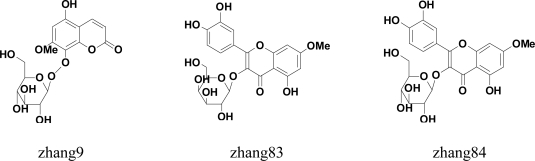

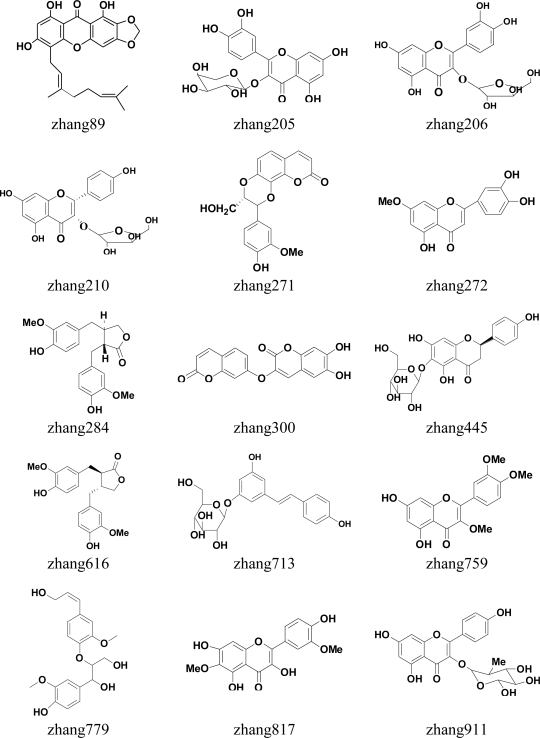
Molecular structure of 18 compounds tested *in vitro*.

Finally, the same hits appearing in the top 20 of both results were chosen. Thus, up to 18 hits (Figure 4) were filtered into the next *in vitro* screening.

### 2.3. *In Vitro* Testing

A total of 18 candidate compounds selected by pharmacophore and docking were examined for their potential to inhibit rhCYP1A2 phenacetin *O*-deethylation activity at a test concentration of 1 μM. The results of *in vitro* testing were listed in Table 2. Five of 18 test compounds (272, 284, 300, 616 and 817) were confirmed to have inhibitory activities to CYP1A2. The model was validated by the *in vitro* experiment where accuracy was 27.8% (5/18).

In this study, 971 herbal compounds were excluded by virtual screen and the exclusion rate was 98.2%. The high exclusion rate for screening larger database was very helpful, since it is not only time-saving and economic, but also helps to improve the success rate of screening test *in vitro*. The high exclusion rate also could help to reduce the number of candidate compounds in CNPD and make the experiment more goal-oriented.

In the present study, two of five test compounds which inhibited CYP1A2 activities were flavonoid compounds. In the early experiment, five of 13 herb compounds which inhibited CYP1A2 activities were flavonoid compounds. The flavonoid compounds accounted for almost 40% (7/18) of the total of all inhibitors. This show that the potential for flavonoid inhibited CYP1A2 has tremendous possibilities. The ability of flavonoids to inhibit CYP1A2 has been extensively confirmed [34,35]. Flavones are present in a wide variety of fruits and vegetables whereas flavones are mainly found in cereals and herbs [35–37]. In some countries flavonoids are commonly used as therapeutic agents and some flavonoids are administered orally or intravenously as drugs [38,39]. However, there was little awareness of the potential for flavonoid interactions with conventional drugs. Some clinical studies have demonstrated that flavonoids can affect the metabolism of other drugs [39–41]. Accordingly, flavonoids’ effect on the activity of CYP1A2 should be given more attention. Other herb compounds were also selected using the pharmacophore model and it was validated that they inhibited CYP1A2 activities *in vitro*. Compound 300 was a coumarins compound and compound 284 and 616 were lignanoids compounds.

## 3. Materials and Methods

**Figure 5.**
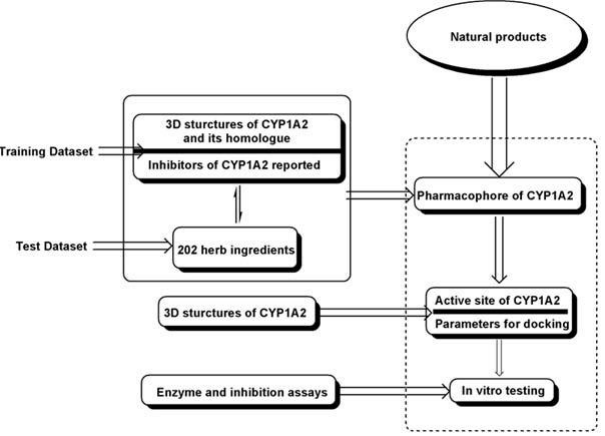
General workflow used in our study.

A tandem workflow is designed to screen the inhibitors of CYP1A2 from natural compounds. This workflow is outlined in Figure 5. Details are listed below: Our analysis was started by constructing the pharmaphore model of CYP1A2. The 3D structures of CYP1A2, its homologues and several reported inhibitors of CYP1A2 were used to test the basic templates. Then up to 202 herb ingredients tested *in vitro* were used to validate and modify the basic templates to derive the most suitable pharmaphore model of CYP1A2. In the following, based on the 3D structures of CYP1A2, an active site for docking was chosen as a complementary process for further screening. Finally, compounds having passed the former filters were tested *in vitro*.

### 3.1. Pharmacophore Generation

The crystal structure of CYP1A2 and its homologue CYP2B4 were retrieved from the Protein Data Bank (PDB code 2HI4 [42] and 2BDM [20], respectively). The endogenic ligands α-naphthoflavone (BHF) and bifonazole (TMI) of those two crystals and several reported inhibitors of CYP1A2 were collected as the template molecules to train the pharmacophore model. 202 herb ingredients tested *in vitro* were used as the test dataset.

The purpose of pharmacophore searching is to facilitate 3D searches of conformation databases using molecule annotations related to ligand-receptor binding (like, H-bond donor, acceptor, hydrophobe, *etc*.). A pharmacophore is a set of structural features in a ligand that are related to the ligand’s recognition at a receptor site and its biological activity. In Molecular Operation Environment (MOE) [43] pharmacophoric structural features are represented by labeled points in space with a conformation of the ligand. Each ligand conformation is assigned a set of annotation points, which is a set of structural features that may contribute to the ligand’s pharmacophore. A database of conformations can be searched with a query that represents a pharmacophore hypothesis. The result of such a search is a set of matches that align the pharmacophoric features of the query to the pharmacophoric features present in the ligands of the searched database.

### 3.2. Docking Procedure

The crystal structure of CYP1A2 (PDB code 2HI4) was used to derive the active site and tune the stable parameters in docking.

**Figure 6.**
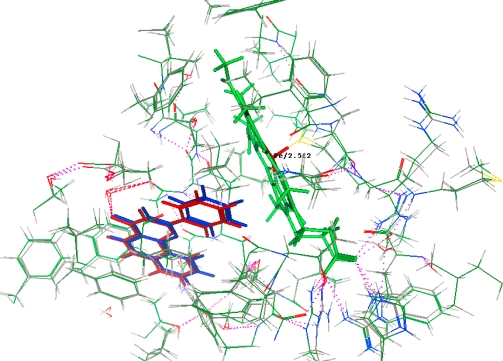
Crystallographic (Red) and docked (Blue) conformations of α-naphthoflavone in the Human Microsomal P450 1A2 receptor site S = −11.1076, rmsd = 0.3061. Note: Hydrogen bond is indicated by the pink dashed line.

MOE-Dock 2008.10 [43] function was used for docking. Ligand placement was performed using alpha sphere triangle matching, with London dG scoring. The top 30 hits were retained and refined using MMFF94X force field energy minimization with Generalized Born solvation model, allowing the receptor residues within 9 Å to relax around the mobile ligand. The active site of receptor’s side chains were tethered with a force constant of 1.0 kcal/(mol Å^2^). Energy minimization was stopped when the root-mean-squared gradient cutoff of 0.0001 kcal/(mol Å) was reached. Final poses were ranked using the London dG method to calculate the free energy of binding. With this set of docking parameters, the crystallographic placement of α-naphthoflavone was accurately predicted in the crystal structure of Human Microsomal P450 1A2 receptor crystal structure, PDB: 2HI4 (Resolution (Å): 1.95) (Figure 6), with a root-mean-squared deviation (RMSD) of 0.3061 Å and a binding energy of −11.1076 kcal/mol.

### 3.3. Enzyme and Inhibition Assays

The basic incubation medium contained 100 mM potassium phosphate buffer (PH 7.4), 50 pmol recombinant human CYP1A2, 25 μM phenacetin and 1 μM active ingredients. The final incubation volume was 200 μL. After 5 min pre-incubation at 37 °C, the reaction was initiated by the addition of NADPH-generating system (5 mM MgCl_2_, 10 mM glucose 6-phosphates, 1 mM NADPH, and 2.5 U per mL glucose 6-phosphate dehydrogenase). Incubations were performed in a 37 °C shaking water bath for 1 h. Reactions were stopped by adding 0.4 mL of ice-cold acetonitrile containing 0.2 mg/L tinidazole (IS). Then 0.6 mL water was added to the mixture, vortex-mixing for 1.0 min and centrifuged at 10,000 g for 5 min, and separation for the supernatants were performed on a ZORBAX SB-C18 column (3.5 μm, 2.1 × 100 mm, Agilent Corporation, MA, USA) and a C18 guard column (5 μm, 4.0 × 2.0 mm, Phenomenex, CA, USA) with an isocratic elution of acetonitrile—0.1% formic acid (35:65, v/v) at a flow rate of 0.3 ml/min for the amount of metabolite (acetaminophen) formed. The column was maintained at 35 °C and the injection volume was 10 μL. The retention times of paracetamol and tinidazole were 1.872 and 3.912 min, respectively. The percent of metabolic activity remaining was calculated by comparing the enzyme activities in the control samples that did not contain active ingredients. The active ingredient demonstrating 10% or greater inhibition at 1 μM was considered to have inhibitory activity. Each set of incubation was carried out with four control samples that contained no active ingredients.

## 4. Conclusions

In this study, we have selected several template molecules to generate pharmacophores. 202 different herb compounds were used as test data to test their inhibitory activity against CYP1A2 *in vitro* and used to identify the best pharmacophore with the highest external predictive power. A highly predictive pharmacophore model was generated with three bifonazole (TMI) in different conformations with sophisticated optimization. A rigorously validated pharmacophore model was then used to screen our in-house database collection of a total of over one thousand herb compounds. 147 hits were filtered out by the selected pharmacophore model and were docked into the active site of CYP1A2. Finally, 18 hits were further filtered and experimentally validated. Five of them were confirmed to have inhibitory activities to CYP1A2, and the accuracy of the model was 27.8%. This study illustrates that the model developed here is efficient for virtual screening of large databases in the identification of CYP1A2 inhibitors or non-inhibitors. Accordingly, the models can play an important role to prevent the risk of e.g., herb–drug interactions through metabolism at an early stage of the drug development process.

## Supplementary Materials



## Figures and Tables

**Table 1.** The results of different pharmacophore models.

**Compounds Used as a Template**	**True Positive Rate**	**True Negative Rate**
BHF, TMI	61.5%	85.2%
BHF, Chrysin and Tanshinone II_A_	69.2%	96.8%
Chrysin, Psoralen and Dracorhodin	61.5%	89.9%
Three TMI in different conformations without optimization	84.6%	77.2%
Three TMI in different conformations with sophisticated optimization	84.6%	86.8%

**Table 2.** Results of *in vitro* testing.

**Number**	**Active Ingredients**	**% of Control Indication Activity**
zhang 9	5-hydroxy-7-methoxycoumarin-8-*O*-d-glucopyranoside	>90%
zhang 83	rhamnetin 3-*O*-β-d-galactopyranoside	>90%
zhang 84	rhamnetin 3-*O*-β-d-glucopyranoside	>90%
zhang 89	1,6,8-trihydroxy-2,3-methylenedioxy-5-geranylxanthone	>90%
zhang 205	quercetin-3-*O*-β-d-arabinopyranoside	>90%
zhang 206	quercetin-3-*O*-α-d-arabinofuranose	>90%
zhang 210	dihydrokaempferol-3-*O*-α-d-arabinofuranose	>90%
zhang 271	5′-demethoxy Daphneticin	>90%
zhang 272	3′-hydroxy-Genkwanin	<90%
zhang 284	(−)-Matairesinol	<90%
zhang 300	Edgeworthin	<90%
zhang 445	Hemiphloin	>90%
zhang 616	(+)-Matairesinol	<90%
zhang 713	Piceid	>90%
zhang 759	3,3′,4′-Tri-Me ether-3,3′,4′,5,7-Pentahydroxyflavone	>90%
zhang 779	(−)-Threo-guaiacylglycerol-8-*O*-4′-(coniferyl alcohol) ether	>90%
zhang 817	Spinacetin	<90%
zhang 911	Kaempferol-3-*O*-α-l-rhamnopyranoside	>90%

Note: Values represent the average of duplicate determinations. Values in italics represent those exhibiting 10% inhibition or greater.
