# Supplementary material for: Novel Natural Inhibitors of CYP1A2 Identified by in Silico and in Vitro Screening
Source: Int J Mol Sci. 2011 May 18;12(5):3250–62. doi: 10.3390/ijms12053250 (PMC3116189; doi:10.3390/ijms12053250)
Supplement: Supplementary file 1 [file ijms-12-03250-s001.pdf]

Supplementary Materials

Table S1. Two prioritizing results by docking (Top 30 from 147).

Index	Result 1 *	Result 2 *
1	zhang84	zhang83
2	zhang205	zhang84
3	zhang83	zhang 210
4	zhang206	zhang 205
5	zhang89	zhang 206
6	zhang911	zhang 817
7	zhang445	zhang 911
8	zhang759	zhang 89
9	zhang271	zhang 779
10	zhang210	zhang 759
11	zhang272	zhang 284
12	zhang 713	zhang 445
13	zhang 300	zhang 271
14	zhang 9	zhang 272
15	zhang 179	zhang 616
16	zhang 1010	zhang 135
17	zhang 876	zhang 381
18	zhang 616	zhang 713
19	zhang 284	zhang 300
20	zhang 779	zhang 9
21	zhang 755	zhang 836
22	zhang 520	zhang 1010
23	zhang 358	zhang 358
24	zhang 132	zhang 850
25	zhang 676	zhang 132
26	zhang 821	zhang 520
27	zhang 817	zhang 28
28	zhang 850	zhang 821
29	zhang 28	zhang 731
30	zhang 381	zhang 755

*: Indicated by the compound IDs named in our lab.

Table S2. Effect of 202 active ingredients of herbs at a Concentration of 1 μM on Rh CYP1A2-mediated phenacetin *O*-deethylation.

Active Ingredients	% of Control Indication Activity (Mean)	Active Ingredients	% of Control Indication Activity (Mean)
Artemisinin	80.7	Icaritin	>90
Chrysin	4.6	Imperatorin	>90
Chrysophanol	77.7	Indirubin	>90
Dracorhodin	21.1	Indigo	>90
Ethoxychelerythrine	88.9	Irisflorentin	>90
Galangin	78.2	Isoferulic acid	>90

Table S2. *Cont.*

Honokiol	33.6	Isofraxidin	>90
Luteolin	87.4	Isopsoralen	>90
Nuciferine	85.5	Isorhamnetin	>90
Osthole	86.8	Jatrorrhizine Hydrochloride	>90
Psoralen	10.8	Jervine	>90
Tanshinone IIA	28.3	Kaempferol	>90
Wogonin	74.6	Kirenol	>90
Aconitine	>90	Liensinine	>90
Alantolactone	>90	Linarin	>90
Aloe-emodin	>90	Linderane	>90
Alpinetin	>90	Linderane_1	>90
Ammothamnine	>90	Liquidambaric acid	>90
Amygdalin	>90	Liquiritin	>90
Andrographolide	>90	Lobetyolin	>90
Anisaldehyde	>90	Loganin	>90
Arctiin	>90	Magnolol	>90
Arecoline	>90	Magnolin	>90
Aristolochic Acid	>90	Matrine	>90
Asiaticoside	>90	Menthol	>90
Astragaloside	>90	Mesaconitine	>90
Atropine Sulfate	>90	4-Methoxysalicylaldehyde	>90
Baicalein	>90	Methyl nonylketone	>90
Baicalin	>90	5-*O*-Methylvisammioside	>90
Barbaloin	>90	Mollugin	>90
Berberine Hydrochloride	>90	Momordin Ic	>90
Bergenin	>90	Muscone	>90
Betaine	>90	Naringenin	>90
Bilobalide	>90	Naringin	>90
Borneolum	>90	Nitidine	>90
Bornylacetate	>90	Notoginsenoside R1	>90
Bufalin	>90	Oleanolic acid	>90
Bufatalin	>90	Oridonin	>90
Caffeic acid	>90	Paeoniflorin	>90
Caffeic acid ethyl ester	>90	Paeonolum	>90
Cantharidin	>90	Palmatine Hydrochloride	>90
Cardamonin	>90	Patchouli alcohol	>90
Carvacrol	>90	Pectolinarin	>90
Catalpol	>90	Physcion	>90
(+)-Catechin	>90	Picroside-II	>90
Chonglou Saponin II	>90	Pinoresinol diglucoside	>90
Chlorogenic acid	>90	Piperine	>90
Cinnamaldehyde	>90	Polydatin	>90
Cinnamic acid	>90	Polygalacic acid	>90
Cinobufagin	>90	Praeruptorin A	>90
Corynoline	>90	Prim-*o*-glucasylcimifugin	>90

Table S2. *Cont.*

Costunolide	>90	Protocatechuic acid	>90
Crenulatin b	>90	Protopine	>90
Crenulatin b_1	>90	Pseudolaric acid B	>90
Curculigoside	>90	Puerarin	>90
Curcumin	>90	Pulegone	>90
Cyclopamine	>90	Quercetin	>90
Cynandione A	>90	Quercitrin	>90
α-Cyperone	>90	Resibufogenin	>90
Daidzein	>90	Rhein	>90
Dehydroandrographolide	>90	Rhodionin	>90
Dehydrocostus lactone	>90	Rhodionin_1	>90
Deoxyschizandrin	>90	Rhodiosin	>90
3,4-Dihydroxybenzaldehyde	>90	Rutaecarpine	>90
β,β-Dimethyl-acry-1alkannin	>90	Rutin	>90
Echinacoside	>90	Saikosaponin A	>90
Emodin	>90	Salidroside	>90
Ephedrine	>90	Salvianolic acid B	>90
(−)-Epicatechin	>90	Sarsasapogenin	>90
Esculetin	>90	Schisantherin A	>90
Esculin	>90	Schizandrin	>90
Eucalyptol	>90	Scopolamine Hydrobromide	>90
Eugenol	>90	Scopoletin	>90
Evodiamine	>90	Scutellarin	>90
Fangchinoline	>90	Sennoside	>90
Ferulic acid	>90	Sesamin	>90
Formononetin	>90	Shionone	>90
Forsythin	>90	Silibinin	>90
Fraxetin	>90	Sinapine	>90
Fraxinellone	>90	Sinomenine	>90
Gallic acid	>90	β-Sitosterol	>90
Gastrodin	>90	Sophoricoside	>90
Geniposide	>90	Stachydrine hydrochloride	>90
Ginkgolide A	>90	Strychnine	>90
Ginkgolide B	>90	Synephrine	>90
Ginkgolide C	>90	Syringin	>90
Ginsenoside F1	>90	Syzalterin	>90
Ginsenoside Rb1	>90	Tectoridin	>90
Ginsenoside Re	>90	Tetrandrine	>90
Ginsenoside Rg1	>90	Tetrahydropalmatine	>90
Glycyrrhetinic acid	>90	2,3,5,4-Tetrahydroxyl diphenylethylene -2-*o*-glucoside	>90
Harpagoside	>90	Thalictrifoline	>90
Hederagenin	>90	Thymol	>90
Hesperidin	>90	Tubeimoside IV	>90

Table S2. *Cont.*

Hupehenine	>90	Typhaneoside	>90
5-Hydroxymethylfurfural	>90	Vanillic acid	>90
Hydroxysafflor yellow A	>90	Veratramin	>90
Hypaconitine	>90	Vitexicarpin	>90
Hyperoside	>90	Vitexin	>90
Icariside I	>90	Vitexin glucoside	>90
Icariside II	>90	Vitexin rhamnoside	>90
